# Nearby, Self-Critical, Ambivalent: Modes of Writing Arab Queerness

**DOI:** 10.1177/15327086251413693

**Published:** 2026-01-17

**Authors:** Lamiae Bouqentar

**Affiliations:** 1 Institute of Women and Gender Studies, 7938 University of Toronto, Toronto, ON, Canada

**Keywords:** queer refusals, writing through encounters, Arab queerness, opacity, self-critical writing, writing nearby, queer ambivalence, fragments

## Abstract

This paper foregrounds what I term *writing through encounters* rooted in the premise of writing as a mode of creation. Encounters are understood as affective intensity generating a change in movement (Ahmed, 2000; Probyn, 2016). Through encounters with the self as well as with Arab/Swana queer immigrants, I articulate self-critical writing practices (Anzaldúa, 1987; Nelson, 2015; Mikkilä, 2024), and writing nearby (Minh-Ha, 1994) as creative and critical writing practices to weave an Arab queerness in a non-linear and fragmentary fashion. By attending to ambivalence as a site of critical inquiry and creative response, it invites a mode of writing that is open, multiple, unfinished, and always in encounters with the entangled worlds it inhabits. In this sense, writing *through encounters* offers novel ways for marginalized voices to reframe their understanding of intersectional identities oscillating between visibility and opacity, migratory experiences, and agencies within the academic landscape.

## Openings

Western epistemologies, combined with the twin forces of colonialism and the Enlightenment have long pursued the fantasy of total legibility—fostering a world in which all that exists must be named, classified and categorized within a Eurocentric grid. This type of deciphering comes with certain violence which, for centuries, has defined the existence of the non-Western world as existing only if it can be disciplined into pre-defined categories. Glissant (1990) argues that the demand for transparency is not an innocent quest for understanding but is rather an imposition of control and domination that coerces non-Western subjects into discursive modes of self-disclosure legible to the dominant order. Racialized and marginalized subjects must perpetually translate themselves, in terms they have no control over. Against this colonial gaze, Glissant (1990) proposes the radical act of “the right to opacity,” the ability to resist the ethnographic fixation of constructing The Other by asserting and reclaiming the sovereignty of blur. Mejdulene Bernard Shomali contends that “In worlds ruled by binaries, by demands to exist within flat categories, the absence of language can feel like the absence of capture” (Shomali, 2024, p. 143).

This tension is further exacerbated by the enduring legacy of colonial representations of the “Orient,” which continue to frame Arab bodies, desires, and cultural expressions within reductive binaries. It is within this context that Edward Said’s foundational critique of Orientalism (1978) gains renewed relevance. His work illuminated the colonial mechanisms through which the “East” has been persistently essentialized, exoticized, and rendered legible only through Western epistemes.

Drawing on Said (1978), several scholars studying queerness in the Arab world have showed how the very categories used to describe sexuality, in the « middle east », emerged through colonial encounters that transformed earlier forms of homoeroticism into the modern, medicalized category of homosexuality (El-Rouayheb, 2005; Gayed, 2024; Massad, 2007). Through her analysis of the starkly different language used by Thomas Edward Lawrence—an Arabist and British spy—to describe homoerotic encounters he observed, Nour Abu-Assab (2017) highlights a telling colonial bias. Lawrence characterized desire between two Arab men as “unspiritual,” while portraying similar relations between British men as “pure.” Abu Assab argues that such colonial practices of translation and interpretation, often mobilized to construct a universal history of gay and lesbian identity, enact a form of symbolic violence that risks reinscribing orientalist assumptions and casting the Arab subject as deviant (Abu-Assab, 2017). Translation operated as a colonial technology, rendering Arab sexualities legible through Western categories while erasing indigenous modes of desire and relationality.

In diasporic contexts, Arab queer writers and thinkers inherit this entangled legacy, grappling with the double bind of resisting colonial discourses while navigating Western frameworks of queerness that often presume universality.

Scholars such as El-Tayeb (2011) and Georgis (2013), have critiqued the epistemic violence entailed in rendering Arab queer lives either invisible or legible only through Western optics of identity politics and visibility, arguing instead for alternative ways of understanding and re-imagining Arab queerness. This decolonial imperative has been carried forward by scholars such as Joseph Massad, whose *Desiring* Arabs provocatively argues that Western gay rights discourses, particularly through what he terms the “Gay International,” replicate colonial logics by imposing Western sexual taxonomies onto non-Western societies. Massad’s controversial claim—that the global dissemination of Western LGBTQ + identities may erase local, culturally embedded practices of same-sex desire—demands a rigorous engagement with the limits and violence of queer universalism. Mack (2017), Georgis (2013) and Gayed (2024) complicate and criticize Massad’s arguments by foregrounding the affective and embodied realities of Arab queer subjects who navigate their identities across multiple registers—discursive, spatial, and political.

These diverse contributions collectively point to a shift in Arab queer theorization: away from a focus on visibility and rights as ends in themselves, and toward a more layered understanding of what it means to write, live, and feel Arab queerness under the weight of colonial residues. Arab queer diasporic theorizing and methodizing, in this light, becomes a performative and affective archive—an attempt to craft languages of selfhood from fractured lexicons, and to reinhabit silenced histories without reproducing their violence.

I expand on these shifts by developing the concept of *writing through encounters* as a potent methodological framework that intertwines feminist, queer, and critical perspectives. Writing through encounters is crucial because it refuses abstraction and instead grounds theory in relational, situated moments where power, affect, and desire are negotiated, allowing Arab queerness to emerge as lived, situated, contingent, and unfinished rather than pre-scripted. Methodologically, it contributes to the body of Arab queer studies by providing a framework that consider intimacy, proximity, and friction in a manner that is accountable to uneven conditions under which encounters occur rather than to stable identities or universal claims. What has been largely missing in the scholarship on Arab queerness is precisely this attention to *encounters* as a site of epistemic production—where meaning is co-constituted, vulnerable, and resistant to both heteronationalist erasure and liberal queer capture.

Encounters, in this sense, are not mere interactions; they are thresholds where subjectivities, histories, and embodied knowledges collide, rupture, and transform (Ahmed, 2000; Moussawi, 2022; Probyn, 2016). They resist containment within binary logics, generating openings for non-normative ways of being, knowing, and relating. To write through these moments is to engage with their complexity, to make space for their affective excesses, and to resist their reduction to normative and colonial logics of understanding.

I argue that a critical writing of Arab queerness demands modes of engagement that are not only ethically attuned to the politics of representation, but that also recognize writing itself as a space of ambivalence, with the latter constituting a productive racialized affect (Berg & Ramos-Zayas, 2015) and refusal of Grand narratives. It is precisely within this contested, fragmented terrain that this paper emerges. A critical feminist queer praxis of writing through encounters thus requires a deep engagement with the multiplicity of affective, historical, and material entanglements that shape lived experiences. It asks: How do the intensities of encounters with theories, the self and others—marked by power, joy, pain, and desire—inform not only what we write but how we write? What does it mean to write in a way that makes space for the opacity and irreducibility of others? By refusing the urge to resolve the ambivalence of encounters, this practice resists the extractive tendencies of dominant writing epistemologies and affirms a commitment to relational, fragmented, situated, and accountable knowledge production.

## Writing Arab Queerness, Ambivalence, and Refusals

To grasp Arab queerness, it is necessary to move beyond static notions of identity that rely on fixed categories and stable definitions. The question is not simply how to “add” queerness to Arabness, or vice versa, but how to grapple with the tensions, contradictions, ambivalence, and relationalities that emerge when these terms encounter one another. In other words, rather than treating identity as a closed system, we might ask: what becomes possible when Arabness and queerness are approached as shifting, intersecting, and destabilizing forces? This orientation allows us to unsettle conventional framings and open up a more dynamic understanding of subjectivity.

Rejecting the task to authenticating queerness in relation to Arabness or locating a fixed Arab queer subject; Sophie Chamas (2024) proposes to “queer” the Arab world and dislocate Western genealogies of queerness, thereby unsettling the dominant notions of queerness and Arabness. Chamas (2024) pushes us to imagine and ask what does it mean to learn and perform queerness as an orientation and becoming that is emerging from, and through Arabness, rather than in counteraction to it? It is within this critical context that Chamas’s (2024) understanding of Arab queerness as a mode of becoming that destabilizes both understandings of queerness and dominant understandings of Arabness offers great insight.

In a similar vein, Sabiha Allouche’s scholarship proposes a particularly nuanced methodological approach to Arab queerness by centering affect, embodiment, and the lived messiness of desire in the context of postcoloniality. Allouche (2019) critiques the binary positioning of the “progressive queer” subject against a “repressive Arab culture,” arguing instead for a decolonial feminist ethics that resists both moralism and exceptionalism while also taking up the task to look inward. Her emphasis on affect as a method—rather than simply an object—of inquiry allows for a deeper engagement with how Arab queer subjects feel, resist, and reimagine themselves in the face of systemic silencing (Allouche & Sayegh, 2020).

Drawing from this line of thought, I understand “Arab queerness” as a frictional ambivalent space, oscillating between non-normative identities, refusal of heteronationalist and neo-orientalist narratives, and dislocation. Centering flux, contradictions and multiplicity generates modes of resistance that refrain from the reductionist and overly simplistic framework of identity focus on singularity to embrace a more complex, fluid, and relational understanding of the Arab queer diasporic experience that underscores the power of ambivalence as a site for constructing new forms of relationality and resistance.

I dreamed of sketching out a queer Arabness at fluctuating temperature, sometimes joyful, sometimes painful. To do so, I had to move away from the grand academic writing, that builds on linear arguments and is characterized by a single form and write this queer Arabness in diasporic contexts differently; by pluralizing writing forms, mixing and multiplying genres and points of entry: Vignettes, poetry, personal narratives and critical writing. Having been inspired by the Arab queer researcher Marc Jahjah, who writes about “hearing voices” from (his) imaginary world (Jahjah, 2021), I found myself writing from and through heterogenous encounters with theories, myself, and others. I had been crafting a collage of unfinished disjointed elements that trace different directions and orientations of a queer Arabness. These forms of fragmental writing allow me to write in a variety of ways, using heterogeneous snippets that are not constrained by a single form that imposes its own codes.

I wanted to bridge creative and critical practices of writing all the while making room for the everyday, the mundane experienced by and in the body.

Futile or political? I, precisely, wanted to get away from this binary, and make room for ambivalence as simultaneously a productive affect and a potent writing practice of the margins.

Ambivalence has been tackled by several racialized queer theorists as it enables the production and imagination of a dwelling space beyond binaries.

Borrowing from Anzaldúa’s “mestiza consciousness,” one can think of ambivalence as a situated type of knowledge dwelling in border crossing, contradiction, and hybrid positions. In Anzaldua (1987), Anzaldúa points out the ways queer and racialized people confront overlapping axes of power: racism, sexism, colonialism, and heteronormativity, all the while managing to resist being reduced to a single identity. Anzaldúa’s ambivalence as a type of inhabiting contradiction without resolution serves as both a limitation and an asset.

In Muñoz (1999), Jose Esteban Muñoz argues for means through which ambivalence is not just tolerated but mobilized. His notion of disidentification is itself ambivalent—as there is no complete incorporation and no total rejection of the dominant culture. Muñoz argues that racialized queer subjects do not passively accept, but strategically and tactically manage and enact alignment with dominant ideologies. Here, ambivalence is political, performative, and crucial for psychic survival. This is even more urgent for the racialized queer subjects that are out of category (Held, 2023) and must frequently read against the grain of their own communities and the dominant queer culture.

Christina Sharpe’s *in the* Sharpe (2016) provides another register of ambivalence, particularly through the affective afterlives of slavery. Rather than looking at grief, loss and mourning as mere privation emotions, Sharpe (2016) proposes to grasp them as collective, racialized affects that endure as both presence and absence. Her reading of the “wake” places Black life in a continuous temporality of loss and survival.

In “*In Defense of Specters: Ambivalent Mourning as Queer Affect*,” Ghassan Moussawi explores the notion of ambivalence not as a problem to be solved, but as a vital enduring affective mode within queer subjectivity, especially those marked by racialization, exile, and marginality, as it is the for Arab queerness. Moussawi (2022) engages with affect theory and queer theory to critique closure-oriented mourning practices that demand resolution and coherence, injunctions that often sanitize racialized queer grief. Using the Derridean concept of “hauntology” and queer futurity by José Esteban Muñoz, Moussawi reframes ambivalent mourning as a sustained spectral attachment that does not assimilate to dominant timelines of affect and liberal linear scripts of healing. Instead, ambivalence, in Moussaoui’s theorization, is a racialized queer affect—an opaque and unruly response to the erasures of modernity and the violence of legibility. In other worlds, rather than accepting Euro-American pathologization of ambivalence, Moussawi (2022) frames it as an act of decolonization, one that refuses the temporal demands of reason, resolution, and rationality. As he notes:

I do not theorize ambivalence as a postmodern feeling nor as pathological (…) I decenter its Euro-American conception in order to think of it as an ongoing emotion that refuses modernity’s obsession with fact-finding, statistics, discrete classifications, truth, and rationality (Moussawi, 2022, p. 88).

Ambivalence, then, becomes a racialized queer survival strategy—simultaneously self-wounding and self-sustaining—that offers space for contradiction, and opacity in navigating the losses and hauntings imposed by intersecting structures of race, gender, sexuality, and empire. It is not a pathology but a modality of living *in spite of*—a resistance to resolution and moving on.

This framework resonates with Sophie Chamas and Sabiha Allouche’s analysis in *“Mourning Sarah Hegazi: Grief and the Cultivation of Queer Arabness,”* where the authors offer a nuanced analysis that foregrounds the ambivalent affective terrain of grief in queer Arab communities. The authors situate Sarah Hegazi’s tragic death not only as a collective mourning event but as a site where conflicting emotions—pain, rage, longing, and desire for visibility—intersect, producing an ambivalence that is deeply racialized and queer. Hegazi’s death, they argue, exposes the tension between personal sorrow and collective identification, revealing how grief operates simultaneously as a private experience and as a communal negotiation of belonging under conditions of systemic erasure (Chamas & Allouche, 2022). Mourning, in this context, emerges as a fraught yet vital practice: it confronts hegemonic heteronormative and patriarchal structures while enacting fragile acts of resistance that oscillate between vulnerability and resilience. By publicly enacting grief, queer Arab communities transform ambivalence into a generative force—one that fosters solidarity, cultivates political consciousness, and produces collective life-making practices that exceed the reach of nation-states. In Chamas and Allouche’s framework, the Arab queer affective experience is thus not simply about survival; it is an ongoing navigation of tension, of relationalities shaped by dispossession, diaspora, and desire, where ambivalence becomes itself a form of resilient witness and transformative praxis.

Articulating ambivalence as a racialized queer affect requires understanding affect not simply as emotion, but as a mode of embodied, historical, and political relation (Berg & Ramos-Zayas, 2015; Blickstein, 2019)—especially under the conditions of racialized and sexualized marginality. Ambivalence, in this context, is not a failure of clarity or conviction but a *survival strategy*, a *critical orientation*, and an *epistemological stance*. It registers the tension between visibility and erasure, longing and refusal, belonging and estrangement that characterizes the lives of many queer subjects of color. Moreover, reclaiming ambivalence as a generative affect, enables to resist both the Western imperative for queer visibility and the Orientalist desire to fix Arab queerness in legible, coherent forms. Ambivalence, here, is not indecision or weakness, but a critical queer practice—an embodied strategy that acknowledges and works through structural contradictions without seeking their resolution. Arab queer ambivalence may therefore manifest as an oscillation between visibility and opacity, shaped by concerns for safety, family, and state surveillance; feelings of longing for home while resisting its norms, as depicted in the following narratives.

## Toward a Feminist, Queer, and Critical Practice: Encountering and Writing Nearby Arab Queer Subjects

In a conceptual response to “the crisis of representation” that loomed in the feminist poststructuralist academic sphere, cultural theorist and film maker Trinh T. Minh-ha (1994)  theorized the practice of speaking *nearby* as an ethical and nuanced perspective to approach marginalized and otherized subjects without attempting to speak for them, or over them. In conversation with Nancy H. Chen, Minh-ha (1994) argues that attempting to “speak for” another person, especially when one holds a position of relative privilege or authority, for example, a researcher with participants, can easily result in appropriation or misrepresentation. *Speaking nearby* is a way of foregrounding the complexity of another’s experience without trying to fully encapsulate it or claiming authority over it. Instead of taking ownership of someone else’s story, the speaker positions themselves *beside* it. Chen and Minh-ha (1994) advocates for an approach that remains proximate but non-invasive, respectful but not appropriative. To *speak nearby* is to acknowledge the impossibility of fully capturing another’s experience while embracing a mode of communication that gestures toward it, creating space for complexity, opacity, and partiality. *Speaking nearby* is often explored and deployed in postcolonial, feminist, and ethnographic contexts, and is particularly relevant to critical conversations on representation, agency, and the ethics of voice in research and writing.

Drawing from Minh-ha’s speaking nearby, I deployed *writing nearby in my thesis*, *as* a writing that seeks to engage in crafting knowledge as an affective, embodied and material process. The research project explored Arab queerness in diasporic contexts as a relational and processual formation, rather than a fixed intersection of identity categories. Instead of positing “Arab” and “queer” as oppositional or incommensurable, the dissertation examined how they co-emerge, interfere, and mutually reshape each other through embodied, situated encounters with media objects, the self and others. Regarding the latter, I proceeded via call for participants on Facebook groups and Instagram pages dedicated to Arab queer communities in Montreal. I have also solicited my personal network, and organizations that serve Arab queer communities in Montreal such as *Helem* and *Mubadarat*. The participants were from different sociodemographic backgrounds and different Arab countries, and all immigrated to Montreal at some point.

Depending on participants’ preferences, some encounters took place in cafés, others during a walk in a park, or via Zoom. These encounters were not identical, not only in terms of location and content, but also in terms of form and temporality. Indeed, there were conversations that generated “three little dots” and unfolded across different temporalities. That is, we met again and stayed in touch. Other conversations had the feel (and lasted the length) of an interview. In this section, I elaborate on encounters with two participants, H’med^1^ and Noor^2^.

When I engaged with participants for my research, the dialogic and affective exchanges that occurred led to moments of shared recognition or divergence. Writing these encounters in a way that is *nearby* allowed me to acknowledge the relational dynamics of power, positionality, and mutual vulnerability while resisting the closure of meaning. Therefore, writing nearby the participants permitted the coexistence of multiple, sometimes conflicting, truths, embracing both the clarity and opacity of lived experiences.

The encounters with the participants were not transcribed or analyzed as such, since the goal was not to represent or capture data as it is usually the case in semi-structured interviews often characterized by extraction. Rather, they inspired writing in fragments. Excerpts of our conversations, snippets of their words composed a writing in fragments where a knot/tension becomes apparent. These encounters are spaces of shared affective intensities—joy, grief, solidarity, and estrangement—that emerge from the shared yet distinct experiences of navigating diasporic Arab/Swana queerness. Such encounters resist the erasure of Arab queer subjectivities by producing counter-narratives that affirm the multiplicity of Arab queer experiences while challenging essentialist representations. Writing through these relational encounters becomes a form of mutual recognition and solidarity, enabling the articulation of Arab/Swana queerness not as a fixed identity but as a dynamic, relational practice of becoming.

For example, the encounter with H’med raised questions about negotiating the intersection of identity while the one with Noor articulated what they termed as “emotional infrastructure” regarding coming out and opacity.

What follow are examples of some of the fragments of encounters with these two participants.


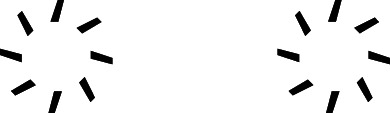


### Emotional Infrastructure and Arab Queer Opacity

Noor used to refer to themselves as an erudite, which carries the weight of knowledge, the weight of ebb and flow—a life lived in many places. Madrid, for an internship with an NGO. Two years in Germany. Eight months in Buenos Aires. Over the course of many years, they have lived in each city as though they were shedding an old skin. To traverse such immense and varying regions is to gather impressions, to see oneself in many different ways, fractured and refracted in different ways from various vantage points. Ethnic and sexual multiplicities shaped their path, led them here—in Montreal, working for an LGBTQIA + rights organization, where identity is a motor for change as much as it is a question.

At 29 this Lebanese immigrant speaks of identity as something that stretches and breathes. Once, in Lebanon, they did not call themselves Arab. It was Montreal that returned Arabness to them, wrapped up in a new meaning, that placed it beside queerness until both felt like facets of the same core. They identified themselves as gay first, then queer, then found language in academic texts that whispered something deeper: non-binary.

“^3^In my white circles, I was told: if your parents do not accept you, then they do not love you,” Noor says, their voice steady over our Zoom call. “I believed that for years. I told them they were backward, that the world had changed, that love was no longer conditional on faith, on gender, on who we choose to hold close.”

“And how did they respond?” I ask.

“They didn’t.”

No scream, no rejection, no contestation—nothing but total silence. As if no words were actually uttered. As if the statement did not happen in the first place.

I know this silence. Empty but sharp enough not to be mistaken for civility. It is a silence that allows for no wonder. We do not abandon you. We do not recognize you. Noor puts it plainly: “We exist in a gray space. Not quite Arab like them over there, not quite queer like them over here.”

Liminality—a familiar dwelling space for those who come from lands where queerness is unspeakable, where existence itself bends under unspoken rules. Identity is never static; it is shaped by the gaze that acts upon it. To be Arab shifts in meaning across contexts. To be queer carries certain expectations, unspoken scripts, assumptions that are framed within a white normative freedom.

*“Postcolonial queer theory has provided me with the tools to heal* ” Noor says.

“How so?”

“I realized Western individualism is not the only option. I would do anything for my family, no matter the tensions. Even if I insist on my truth, I will not erase theirs. And honestly? We don’t have the emotional infrastructure to hold that kind of conversation.”

The phrase lingers long after our conversation. *Emotional infrastructure.*

Infrastructure—the hidden veins of a city, the foundations that absorb impact, the structures that allow movement, resilience, repair. It is what permits a truth to land—a port, what permits a body to hold itself under the weight of its own intricates.

It is never only personal; it is collective, interwoven into kinships, histories, and social fabrics. A society’s capacity to endure disorientation, to step outside the straight line and take a queer leap (Ahmed, 2006) is a question of presence—or absence—of this impact space.

I think of my own parents. Of the many years that I held back my unvoiced confession. Not in words, but in the margins of my research papers, upon alighting with friends in Rabat to whispered conversations at 3 AM, and in every other place where I let myself, really, come to be. I, too, believed that the absence of the infrastructure elaborated my opacity. That if a person is shaped by their social world—by politics, by history, by unspoken rules—then emotional infrastructure must be systemic, must be a thing beyond the control of those we love.

But does that mean I have found peace?

The answer, like Noor’s journey is ever shifting.

Elusive as breath.


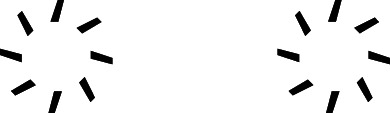


### Binaries

Lida’s message seemed to arrive like some kind of an invitation for something that I had to do-a gay Moroccan eager to take part in my research. H’med, Once a worker in Moroccan tourism, now a man of numbers in the heart of Montreal’s banking district. Fifteen years had passed since his migration, but time had been kind to him—slicked-back hair, a meticulously trimmed beard, a sheer pink shirt draped over a sculpted chest. At fifty-three, he carried a light freshness of someone who defied the arithmetic of age.

He deliberately called himself a *gay Arab-Muslim*—with the precision of someone who has rehearsed his self-definition. *“I prefer gay over queer. ‘Queer’ is vague, trendy. I don’t even know what it means, but hey... Whatever. Yes, I’m a practicing Muslim. I pray five times a day, and it is important to me*.*”*

H’med was one of those who left because staying was untenable after his cousin outed him.

His bodily movements were almost choreographed, hands slicing the air with a thought, like he was putting his life in boxes, separating, organizing, ensuring that everything was neat, that nothing tangled. He exuded order, a man who had mastered his own contradictions and strived for balance.^3^

His Canadian passport granted him the privilege of going back and forth to Morocco, yet the home-country remained a distant shore, blurred by estrangement. A sister unwilling to accept his gay identity, a home emptied of parents, a past that no longer beckoned. *“I vacation where the sun feels good—the south, for example.”* And yet, Morocco held him still, tethered in ways distance could not sever. From across the ocean, he envisioned an online project that aimed to help gay men in the rural parts of his ancestral country—gay men who had been told their desires were unnatural. *“I want them to know they are not alone. To educate them about protection, about support. I can do that from here, where I have the privilege to speak.”*

While in Montreal, Morocco still lingers in his rituals. Sundays and sometimes Fridays were spent in Laval in company of his Moroccan heterosexual friends. Afternoons at Le Petit Maghreb, who according to him serves the best couscous in the city, where nostalgia was served in clay dishes. Evenings in the Gay Village^4^, where another version of himself emerged, unfettered, moving between bars, between selves.

It was amazing to see how ordered yet controlled his existence was: The straight world of Laval. The queer world of the Village. The diasporic world of Le Petit Maghreb. A careful dance of separations.

“That seems like a very meticulously designed arrangement,” I said.

“*That is true but is not the whole truth*” he responded with a chuckle.

*“Oh? How so?*”

*“Well, ironically, since moving here—fifteen years now—I find it harder to be in a relationship than I did in Morocco. There, I had to be careful, always plan my every move. Yet, I managed to date and love men. But here... here, it’s just harder. I guess you might think it’s strange, isn’t it?”*

I didn’t find it strange, only revealing. It unsettled the assumption that gay love should come easily in a land that prides itself on its queer freedoms. H’med’s words echoed Momo’s, another participant I encountered who had confessed under the weight of an evening’s fatigue, *“In Tunisia, relationships felt simpler. Here, something always feels off.”*

It was a quiet rebellion against the fantasy of the West as a *gay paradise.* For those who arrived as immigrants, even after becoming citizens, there were codes left unmastered, languages of intimacy not yet fluent.

In H’med’s rehearsed choreography, I wondered: Did his friends in Laval know he was gay? Did his friends in the Village know he was a practicing Muslim?

*“I don’t lie to them,”* he said. *“It’s just that my Laval world wouldn’t mix well with my Village world ... So, I keep them separate.”*

But how porous were the borders? How much of the self passes through, regardless of how fine the line?

H’med’s story was much like my own, a life lived in a fragile balance, having to navigate the existence of parallel selves, negotiating spaces that would never truly coincide. I often visited Morocco, but my stays there were purposely staged. Queer love stories omitted, and silences maintained.

To live in the in-between is a way of making a home in the interstice. To learn the art of withholding and revealing, to exist in fragments—each piece whole in its own form, yet never entirely reconciled with the others.

These encounters with the participants have also pushed me to reckon with my own entanglement in the very cartographies of Arab queerness I am trying to map.

## The Arab Queer Self: a Complex Terrain of Creative and Critical Engagement

Several feminist theorists (Haraway, 1988; Harding, 2009; Hooks, 1991) have highlighted the socio-historical construction of scientific discourse and its supposed neutrality, transcendence, universalization and veracity.

Indigenous and black studies approaches have articulated an epistemology and methodology of refusal in which the (colonial) terms with which certain theoretical, political and colonial conversations are posited are refused (Mengesha & Padmanabhan, 2019; Museus & Wang, 2022; Simpson, 2007). Atallah and Dutta (2022) articulate a practice of refusal with a decolonial approach that performs refusal as resistance to disciplinary shackles that veil the colonial dynamics of a certain division and organization of knowledge (Atallah & Dutta, 2022). To engage in practices of refusal is to engage in an insistence on imagining and doing (writing) differently, to bring into existence the possibility of new possibilities (Lear, 2001). Not only dreaming of new possibilities, but embodying them, performing them through certain practices of writing, expression and the making of new objects of study.

Mejdulene Bernard Shomali, author of *Between Banat: Queer Arab Critique and* Shomali (2023), has argued that an epistemological and methodological posture of refusal has the potential to emerge as an affirmative gesture that generates queer Arab horizons (Shomali, 2023).

Building on Shomali’s insight that refusal can serve as an affirmative horizon for queer Arab critique, I offer writing through encounters with the self, a parallel mode of inquiry—one that transforms refusal into a method of narrating and theorizing experience. Drawing from contemporary creative-critical writing practices (Mikkilä, 2024), that are genre-defying practices and unsettling the conventions of academic discourse in the late 20th century: such as autotheory, life writing, fictocriticism, autofiction, creative non-fiction and other hybrid writing forms that while each distinct, have in common a resistance to mastery and unitary discourse (Mikkilä, 2024; O’Sullivan, 2024); *writing through encounters with the self* subverts the presumed boundaries between the personal and the theoretical, the aesthetic and the analytical. This mode of inquiry neither privileges subjective confession nor disembodied critique but insists on their intertwinement. It positions the act of writing as *an encounter*—at once with one’s lived experience and with the conceptual frameworks through which that experience becomes legible—thereby foregrounding the productive tensions between creative expression and critical reflection.

I encountered this genre-bending writing while reading “The Argonauts” (2015) by cultural theorist and literary critic Maggie Nelson. Nelson’s fragmental prose, featuring a body of theory cited in the margins, generated a curated intimacy and relational self through which queer perspectives on issues of gender, family-making are interwoven in a non-linear fashion.

Soon after, I found out that Numerous authors in queer studies have engaged in types of critical self-writing, such as the likes of Preciado (2013) or Wark (2023) among others, making the self as not only a space where the personal materializes but also where theoretical reflections emerge. Indeed, by centering the personal as a site of theoretical engagement; the self, in these feminist queer writing practices, becomes both the subject of analysis and the lens through which broader structures of power and oppression are interrogated. This mode of inquiry becomes a feminist, queer, and critical practice that not only critiques existing structures but also envisions new possibilities for knowledge production, identity theorizations, and community-building (Zwartjes, 2019). It also generates not a stable or autonomous entity but a site of tension, multiplicity, and becoming, making room for a cross-border writing that raises the question of what is theory? How do we engage and live with theory?

The work of the authors cited above have deeply challenged the form of academic writing and critique while also unsettling the foundation of the poststructural subject in its presumed heterornormativity and asserting a queer way of blurring life and theory (Fournier, 2021). Yet, these canonical authors of auto-queer writing fall short from engaging critically with their own *white* bodies, reproducing instead what Sara Ahmed calls as a “disappearance of race” (Ahmed, 2006). For whiteness, writes Sara Ahmed “is an orientation that puts certain things within reach […] not just physical objects, but also styles, capacities, aspirations, techniques, habits” (Ahmed, 2006, p. 154). For theorists such as Nelson or Preciado, the whiteness of the bodies that disappears from their autotheoretical endeavor can afford to not be talked about because they are “bodies-at-home” to quote Ahmed. Moreover, Ahmed (2006) understanding of whiteness as an orientation poses the issue of who gets the legitimacy to write and theorize their stories and their lived experiences. That is, who has access to such a craft of creative practices. In a response to Maggie Nelson’s lecture *“The Forms Things Want to Come As*” Elahe Haschemi Yekani notes that within the Black radical tradition and queer of color critique, the displacement of subjectivity emerges as inseparable from the lived realities of racialization—realities that not only unsettle conventional formations of selfhood but also call into question, at the most fundamental level, the very terms through which inclusion within the category of the human is determined and enacted (Yekani, 2025).

The queer diasporic Arab subject—positioned at the intersection of postcolonial displacement and queer non-normativity—mobilizes the “I” not as a gesture of liberal individualism, but as a fractured, politicized subjectivity that resists both orientalist representation and heteropatriarchal nationalisms. Therefore, enabling the researcher to navigate and articulate the multiplicities of Arab/Swana queer identity while challenging dominant epistemologies. This might involve narrating moments of alienation, resilience, or desire and joy as a way to theorize broader phenomena like heteronormativity, cultural dislocation and resistance, or the coloniality of race and gender. These personal narratives not only underscore the researcher’s lived experiences but also challenge dominant epistemologies that have historically excluded and marginalized such voices.

The following are examples of critical self-writing fragments with regards to my coming out and the nexus visibility-opacity.


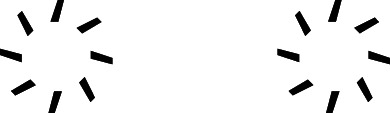


### What’s Your Story?

Writing these lines on my (non) coming out, on my queerness, on the entanglement of Arabness with visibility and opacity, has been an exercise in hesitation, erasure, postponement, and return. It has taken multiple attempts—beginnings and endings are still askew.

At times it has seemed to me that the only story of importance is not the one about myself, that it is rather useful to focus on the narratives of other people, to bear witness with care and proximity. And yet, the impulse to articulate, to inscribe my experience onto language, remains—a means of survival, of resistance, of sketching an alternative horizon for Arab/SWANA queer communities.

But when the decision to write was finally made, the question of beginnings became elusive. *When did you know? When did you become queer?* These inquiries, meant to contain and define, have often unsettled me, rendering the act of narration an uneasy task. How does one structure a story that resists linearity? How does one honor its multiplicities—the layers of analysis, the changes in understanding, the sedimented weight of lived experience?

What does it mean to come out? To perform, to inhabit, to negotiate, to navigate it? The desire to name oneself without fear remains. It is tempting to assume that, having long engaged in autoethnographic and autotheoretical inquiry, narrating my own experience would come naturally. And at times, it does. At other times, it feels insurmountable.

If writing, as Deleuze (1975) suggests, is an act of mapping territories yet to come, it is also a practice of deterritorializing the major. How does one write, then, their own silence, their non-coming out, without reducing it to a form of therapy, and without framing it as a trauma to be triumphed over?

The intersection of postcolonial and decolonial queer theories has brought me into the company of a community of racialized queer scholars who critique the imperative to come out—an imperative that has always felt limiting to me. This line of critique makes visible the ways coming out, as an essential dimension of the so-called sexual liberation paradigm, is severed from the collective and culturally nuanced understandings of freedom that are so sorely needed (Mahmood, 2009; Nelson, 2021). In acknowledging this tension, I understand my experience not as an absence, but as a refusal, an ambivalent and (un)muted form of resistance which disrupts the main story of absence, and the nexus of visibility and belonging.


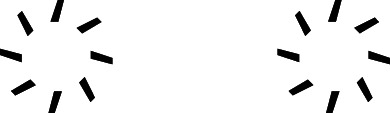

Our Colors

Arab and queer

In their eyes

It’s an oxymoron

In ours, it’s a joy.

They want us to see

In black and white,

But we can only see

In color.

Through that reddish light that shimmers,

The fragments reassemble

And allow us to emerge in harmony.

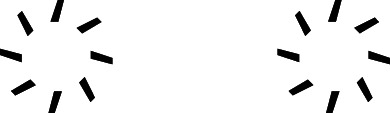


### Backwash

I am sitting in a café seaside with my uncle, aunt, cousin, and my parents. I feel the scent of the air full of salt and mint as a pleasant addition to the warm sun caressing my face, making a warm December day in oh so beautiful Rabat. As I lift my cup of tea, its warmth curling around my fingers, my mind drifts to Montreal, where winter bites and breath turns to frost. How fortunate I am to be here, beneath this home-sun, sipping this tea that only ever tastes right in the presence of my [Moroccan] family. To be enveloped in conversation, in laughter, in the simple murmurs of daily life—things I often dismiss, sometimes even resent, but here, today, they feel precious.

My aunt clasps my hand, her touch infused with tenderness. “*I miss you so much”* she whispers, pulling me into an embrace that carries the weight of distance, of time. In this moment, I feel it—the kind-heartedness of a Mediterranean-Arab home, a love that is expansive, unreserved. A belonging I both crave and escape. Between sips of tea, they pose the inevitable questions:

*“Why don’t you come back? It’s so cold over there.”*

*“You speak English and French; you’d find a good job here.”*

The waves murmur against the shore, filling the silence before I answer. I explain—softly, carefully—that returning is not as simple as they believe. That I cannot simply drop my life there and weave a new one here.

*“Why not?”* my aunt challenges. *“You’re not married, you have no responsibilities.”*

And just like that, I remember. I remember why I left. The quiet insistence that a woman’s worth finds its fullest form within the space of marriage, within the grasp of norms. I swallow my discomfort, let it settle like residue at the bottom of my cup. I have learned—am still learning—that not every battle must be waged. That sometimes, silence is strength.

The waves lull me back into quietude, my gaze tracing their rhythmic retreat. But then, a disturbance—a street vendor weaves through the tables, newspapers in hand. He stops at ours, unfolds the front page of the newspaper الصباح, its bold headline cutting through the sunlight:

*“Arrest of a married female judge caught kissing another woman.”*

The words hang between us like a blade.

*“This sickness is spreading, it’s the West corrupting us”* my uncle mutters.

*“ But if she was born this way, what can we do? It’s not for us to judge.”* Said my mother.

*“Still, she was unfaithful” my father jumping into the conversation.*

*“I don’t think they should have outed her. No one has the right to out someone”* my young cousin says, her voice softer.

*“Outed? What does it mean?”* my uncle scoffs.

Like waves crashing against rocks, their voices echo, swell then crash into one another. It’s my turn to speak. My turn to breathe. I make sure I do not move; I do not make a sound. But my heart beats faster. My breath becomes shallow.

The sea, once my refuge, now mirrors my unrest. I tell myself it is only words, only opinions. That they are not speaking of me. But anxiety is insidious, slipping through cracks, constricting, suffocating.

And in my mind, I scream:

*“Do you want to know why I left? I left because of this. Because of your judgments. Because there is no room for difference here. That is why I left!”*

I picture myself running, running until I lose my breath, until silence swallows me whole. My hands fumble through my bag for relief. I can’t find my anxiety medication. I close my eyes, inhale deeply, wait for the storm to pass.

A line floats into my mind, a whisper from Sofia Samatar’s book “Opacities: on Writing and the Writing life*”: “You know your culture is happening to you when you find it impossible to speak”* (Samatar, 2024, p. 58).

And so, I say nothing.

I focus on the sea, its waves, and let them carry away the words I cannot.

## Closing

Writing through encounters illustrates that for Arab queer lives, informed by colonial pasts, migratory displacements, and resistant collectivities, calls for a mode of inquiry that engage with relational complexity and embodied affective charge of the situated encounter.

This critical creative approach highlights a reflexive grappling with the layered positionalities of Arab queer subjectivity, as a site of negotiation through the slippages of visibility, opacity, and cultural proximity, while also troubling the distinctions of the researcher and the researched. Therefore, the two practices of writing, nearby and self, are entangled, constituting an approach that consolidates ambivalence as a racialized queer affect—the simultaneous pull of belonging and alienation, vulnerability and resistance. Through this writing practice, these ambivalent encounters generate a mode of inhabiting the contradictions of racialized queer life while producing knowledge that is situated, relational, and transformative.

Taking up writing as inquiry thus foregrounds what is politically and epistemically at stake when the act of narration becomes a method for theorizing Arab queer life. Writing becomes not merely a reflection of experience but a performative site where power, desire, and precarity converge, raising the question: how does the labor of crafting narrative itself reshape the conditions under which marginalized subjects can appear, speak, and be heard? By situating writing as a generative yet fraught practice, this approach underscores the risks of exposure, the ethics of representation, and the transformative possibilities of imagining otherwise. In doing so, it insists on a mode of inquiry that attends to the tensions between safeguarding opacity and cultivating relational legibility, allowing writing to function simultaneously as method, critique, and world-making gesture.

## Notes

1. H’med (May 2023). Remote conversation conducted via the Zoom platform, which more closely resembled an interview, characterized by a predominantly question-and-answer format, in which I occupied the position of researcher and he that of respondent. The conversation lasted 60 minutes.

2. Noor (July 2023). Conversation conducted on Zoom, lasting roughly 90 minutes. Noor possesses theoretical knowledge of queer and Arab issues; for example, they are familiar with terms such as “racialized,” “BIPOC,” and “SWANA.” During my conversation with Noor, I had the impression of engaging with a colleague around issues of coming out and Arab queer intersectionality, both in local contexts and in those of the country of origin.

3. Throughout this article, I use Sabon Next Italic to highlight participants’ voices.

4. Montreal’s LGBTQIA + neighborhood.
